# Overview of research progress on the association of dietary potassium intake with serum potassium and survival in hemodialysis patients, does dietary potassium restriction really benefit hemodialysis patients?

**DOI:** 10.3389/fendo.2023.1285929

**Published:** 2023-11-29

**Authors:** Zuoya Sun, Jian Jiao, Gang Lu, Ruihong Liu, Zhuo Li, Yi Sun, Zhiyuan Chen

**Affiliations:** ^1^ Department of Family Medicine, The University of Hong Kong-Shenzhen Hospital, Shenzhen, China; ^2^ Department of Gastroenterology, Shandong Provincial Hospital Affiliated to Shandong First Medical University, Shandong First Medical University, Jinan, Shandong, China; ^3^ Department of Nephrology, Beijing Huairou Hospital of University of Chinese Academy of Sciences, Beijing, China

**Keywords:** hemodialysis patients, dietary potassium intake, serum potassium, dietary patterns, dietary potassium restriction

## Abstract

For the general population, increasing potassium intake can reduce the incidence of cardiovascular and cerebrovascular diseases. However, since hyperkalemia is a common and life-threatening complication in maintenance hemodialysis patients, which can increase the risk of malignant arrhythmia and sudden death, the current mainstream of management for hemodialysis patients is dietary potassium restriction in order to prevent hyperkalemia. Hemodialysis patients are usually advised to reduce dietary potassium intake and limit potassium-rich fruits and vegetables, but there is limited evidence to support this approach can reduce mortality and improve quality of life. There is still no consistent conclusion on the association between dietary potassium intake and serum potassium and survival in hemodialysis patients. According to the current small observational studies, there was little or even no association between dietary potassium intake and serum potassium in hemodialysis patients when assurance of adequate dialysis and specific dietary patterns (such as the plant-based diet mentioned in the article) are being followed, and excessive dietary potassium restriction may not benefit the survival of hemodialysis patients. Additionally, when assessing the effect of diet on serum potassium, researchers should not only focus on the potassium content of foods, but also consider the type of food and the content of other nutrients. However, more large-scale, multi-center clinical trials are required to provide high-quality evidence support. Besides, further research is also needed to determine the optimal daily potassium intake and beneficial dietary patterns for hemodialysis patients.

## Introduction

Maintenance hemodialysis (MHD) is one of the primary alternatives to prolong the survival time and improve the quality of life of patients with end-stage renal disease. As renal function declines, the incidence of hyperkalemia progressively rises, which increases the risk of arrhythmias and sudden death (1–4). MHD patients mainly regulate potassium metabolism in the body through hemodialysis, restriction of potassium intake, potassium-lowering medications and potassium excretion by residual renal function (5, 6). Despite the fact that dialysis can effectively remove potassium ions from the body, the increase in pre-dialysis serum potassium caused by extended dialysis intervals poses a grave threat to patients’ lives. Interventions to reduce pre-dialysis serum potassium may effectively reduce the mortality of hemodialysis patients (7). According to a study of 55,183 patients in the Dialysis Outcomes and Practice Patterns Study (DOPPS) multinational cohort, those with pre-dialysis serum potassium levels between 4-5.5 mEq/L had the lowest risk of mortality, whereas those with levels >5.6 mEq/L had a significantly higher risk of both death and arrhythmia (8). In their study of the influence of serum potassium and dialysate concentration on hemodialysis patient survival, Kovesdy et al. discovered that when pre-dialysis serum potassium was in the range of 4.6-5.3 mEq/L, the survival rate was highest, and with adequate nutrient intake, dietary strategies should be combined to prevent substantial fluctuations in serum potassium (9). Hemodialysis alone is insufficient to maintain normal serum potassium levels, and studies have indicated that controlling dietary potassium intake plays a crucial role in achieving stable serum potassium levels (10). A systematic review of changes in serum potassium (11), chronic kidney disease (CKD) progression, and mortality in patients with CKD following a low-potassium diet versus an unrestricted diet indicates that dietary potassium restriction, compared with higher potassium intake, may lower serum potassium in patients with normokalemia, and they discovered no evidence that dietary restriction is associated with decreased all-cause mortality in patients with CKD stages 3-5. For hemodialysis patients, dietary potassium restriction is currently the mainstream of management in order to prevent hyperkalemia, the latest Nutrition KDOQI Guidelines (12) propose an individualization of the dietary potassium intake recommendation. Other clinical practice guidelines in the field of renal nutrition recommend reducing dietary potassium intake to 2–2.5 g/day for patients undergoing hemodialysis, but evidence supporting such restriction independent of food source to improve morbidity, mortality, and quality of life in hemodialysis populations is limited and not supported by rigorous randomized controlled trials (11, 13–18).

Adequate and satisfying food is an essential human need and enjoyment before security, belonging, self-esteem, and self-actualization are satisfied. Moreover, for the general population, increasing dietary potassium intake can not only lower blood pressure (19–25) and the risk of cardiovascular and cerebrovascular disorders (26–30), but also have other protective effects, such as anti-inflammatory, anti-fibrosis and anti-oxidation effects, improve endothelial function and prevent atherosclerosis (31).

However, to prevent and manage hyperkalemia, many medical institutions have incorporated dietary instruction for hemodialysis patients advising them to limit their consumption of potassium-rich fruits and vegetables (32), more than half of hemodialysis patients report feeling deprived, feeling that the prescribed diet is bland (33). In addition, dietary restrictions that are too stringent might cause malnutrition and lower the quality of life for hemodialysis patients (34, 35).

**Figure 1:**
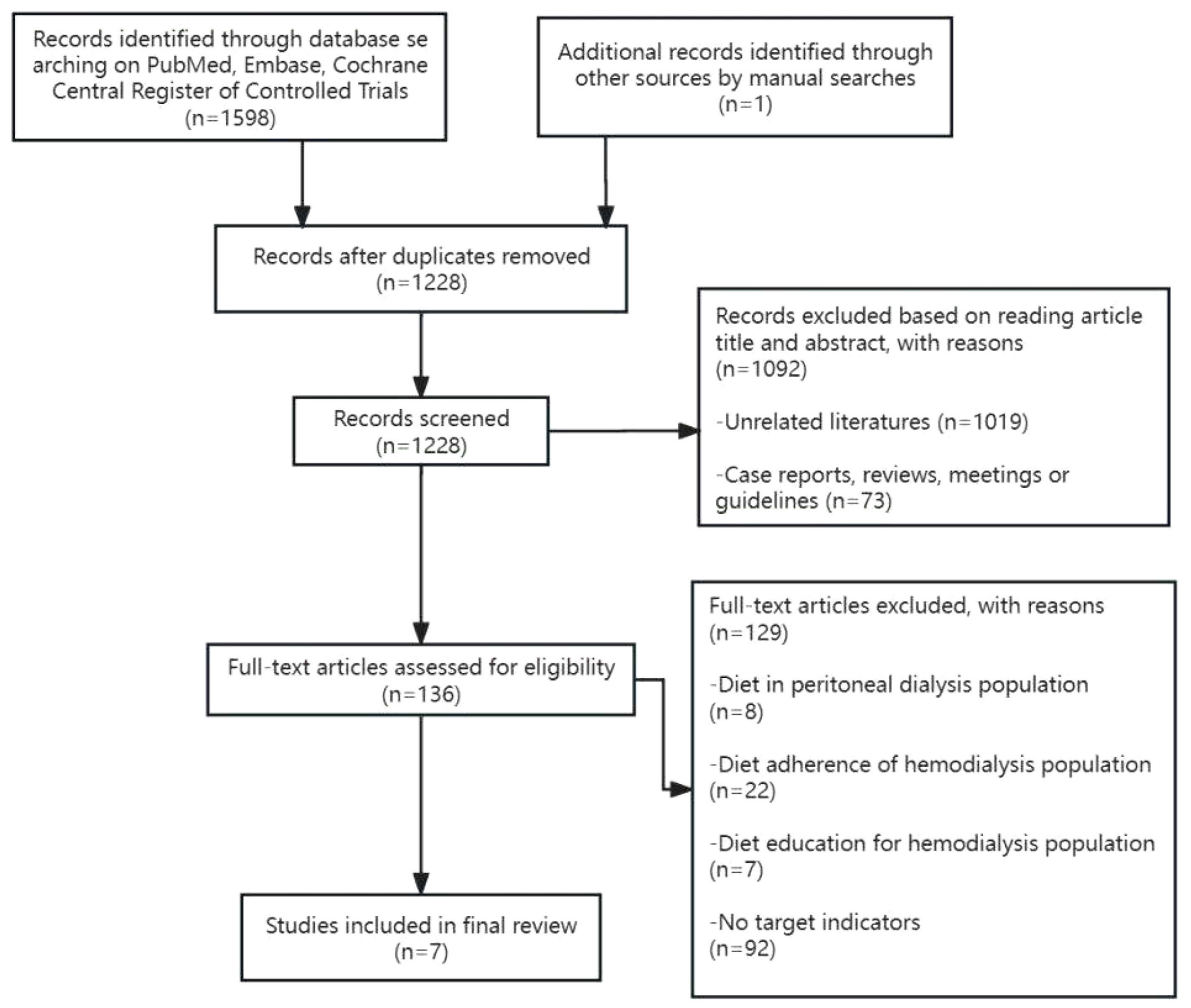
Study selection.

Currently, there is no evidence from observational studies regarding the optimal daily potassium intake for hemodialysis patients, nor is there a consistent conclusion regarding the relationship between dietary potassium intake, serum potassium, and survival rate. This article is a narrative review to illustrate the relationship between dietary potassium intake and serum potassium and survival in hemodialysis patients by comparing the changes in serum potassium, the occurrence of hyperkalemia, and the impact on survival with dietary potassium restriction and non-dietary potassium restriction. We searched PubMed, Embase, and the Cochrane Central Register of Controlled Trials from inception to June 12, 2023, using the terms “Dietary Potassium or Potassium, Dietary and dialysis, renal disease, or kidney failure.” All studies titles and abstracts were examined by two independent reviewers. A third investigator was consulted in the event of a disagreement to enable consensus-building. Finally, seven studies were included for analysis (**Figure 1**).

## Overview of studies on the relationship between dietary potassium intake and serum potassium and its effect on hemodialysis patients

(The basic characteristics and outcomes of the clinical researches included in our study are shown in **Table 1**).

**Table 1:** Characteristics and the effects of dietary potassium intake on serum potassium of 7 clinical researches in hemodialysis patients.

Study/year	Country	Study design	No. Patients	Study duration	Dietary potassium Intake	Serum Potassium	Outcome
Khedr.^[36]^ 2009	Cairo, Egypt	Observational study	HD patients (n=400)	Not mentioned	The patients were classified into three groups: potassium-rich diet (n=214), low potassium diet (n=11), and average diet (n=129), but the specific intake was not mentioned.	Pre-dialysis serum potassium (low-potassium diet):4.309±0.321 mEq/L Pre-dialysis serum potassium (average diet): 4.941±0.376 mEq/L Pre-dialysis serum potassium (potassium-rich diet): 5.691±0.462 mEq/L	Patients on the potassium-rich diet had higher mean serum potassium at pre-dialysis, post-dialysis, and pre-next session than those on the average potassium diet and those on the low-potassium diet (p < 0.01).
Noori.^[37]^ 2010	the South Bay Los Angeles, California, United States	Observational study	HD patients (n=224)	63 months follow-up	Quartiles of potassium intake: Quartile 1: 879±161 mg/d (n=56); Quartile 2: 1342±109 mg/d (n=56) ; Quartile 3: 1852±217 mg/d (n=56); Quartile 4: 3440±969 mg/d (n=56)	Serum potassium (Quartile 1 of potassium intake): 5.1±0.5 mg/dl; Serum potassium (Quartile 2 of potassium intake): 5.0±0.5 mg/dl; Serum potassium (Quartile 3 of potassium intake): 5.1±0.5 mg/dl; Serum potassium (Quartile 4 of potassium intake): 5.2±0.5 mg/dl	potassium intake was positively correlated with the dietary energy, protein and phosphorus intake, and also marginally (r=0.14, p<0.05) with predialysis serum potassium.
Bernier-Jean.^[38]^ 2021	Europe (France, Germany, Italy, Hungary, Poland, Portugal, Romania, Spain, Sweden, and Turkey) and South America (Argentina)	Prospective, multinational study	HD patients (n=8043)	4 years follow-up	Quartiles of potassium intake: Quartile 1: 1.84 g/d (n=2011); Quartile 2: 3.00 g/d (n=2011) Quartile 3: 4.19 g/d (n=2010) Quartile 4: 7.18 g/d (n=2011)	Serum potassium (Quartile 1 of potassium intake): 4.9 mEq/L; Serum potassium (Quartile 2 of potassium intake): 5.0 mEq/L; Serum potassium (Quartile 3 of potassium intake): 5.0 mEq/L; Serum potassium (Quartile 4 of potassium intake): 5.1 mEq/L;	1. Dietary potassium intake was not associated with allcause mortality (per 1 g/d higher dietary potassium intake: hazard ratio, 1.00; 95% CI, 0.95 to 1.05). 2. Potassium intake was not significantly associated with serum levels (0.03; 95%CI, -0.01 to 0.07 mEq/L per 1 g/d higher dietary potassium intake) or the prevalence of hyperkalemia (≥6.0 mEq/L) at baseline (odds ratio, 1.11; 95% CI, 0.89 to 1.37 per 1 g/d higher dietary potassium intake). 3. Hyperkalemia was associated with cardiovascular death (hazard ratio, 1.23; 95% CI, 1.03 to 1.48). 3. Higher dietary potassium intake was associated with a lower risk of noncardiovascular mortality but not of all-cause or cardiovascular mortality. 4. The intake of fruitsand vegetables was associated with a lower risk of all-cause mortality.
Ramos.^[39]^ 2021	Mexico City, Mexico	Cross-sectional study	HD patients (n=117)	Not mentioned	Potassium intake in the normokalemia group: 1.7 g/day (n=58); Potassium intake in the hyperkalemia group: 1.6 g/day (n=59);	Serum potassium in the normokalemia group: 4.3±0.5 mEq/L (n=58); Serum potassium in the hyperkalemia group: 5.8±0.6 mEq/L (n=59)	1. No association was found between serum potassium and potassium intake (r=-0.06, P=0.46). 2. Dietary potassium was not associated with serum potassium or hyperkalemia in HD patients.
Garagarza.^[40]^ 2022	Portugal	Observational, cross-sectional, multicenter study	HD patients (n=582)	1 year follow-up	Mean dietary potassium intake: 2465±1005 mg/day	Mean serum potassium: 5.3 ± 0.67 mEq/L The lower potassium intake group(≤3000 mg/day): 5.2 ± 0.69 mEq/L(n = 418); The higher potassium intake group(>3000 mg/day): 5.4 ± 0.60 mEq/L(n = 126)	1. The potassium-rich DASH dietary pattern was not associated with elevated serum potassium levels in HD patients. 2. High adherence to the DASH dietary pattern predicted lower serum potassium levels (P=0.004).
Narasaki.^[41]^ 2021	Southern California, United States	Prospective cohort study	HD patients (n=415) Tertile1 (dietary potassium intake of <903 mg/day, n=138) Tertile 2 (dietary potassium intake of 903-<1631 mg/day, n=138) Tertile 3 (dietary potassium intake of 1631- 7411 mg/day, n=139)	3 years follow-up	Tertile 1: 543±221 mg/day; Tertile 2: 1234±198 mg/day; Tertile 3: 2606±1166 mg/day	Tertile 1: 4.9±0.6 mEq/L; Tertile 2: 4.9±0.5 mEq/L; Tertile 3: 5.0±0.5 mEq/L	1. There was a monotonic increase in death risk with incrementally lower levels of dietary potassium intake. 2. No differences in serum potassium among groups with dietary potassium intake.
Gonza´lez-Ortiz.^[42]^ 2021	Mexico City, Mexico	Observational single-centre cohort study	HD patients (n=150)	1 year follow-up	Median dietary potassium intake in the low HPD adherence group: 993 mg/1000kcal/day(n = 43); Median dietary potassium intake in the moderate HPD adherence group: 1039 mg/1000kcal/day(n = 53); Median dietary potassium intake in the high HPD adherence group: 1094 mg/1000kcal/day(n = 54)	Median serum potassium in the low HPD adherence group: 4.9 mmol/L(n = 43); Median serum potassium in the moderate HPD adherence group: 5.1 mmol/L(n = 53); Median serum potassium in the high HPD adherence group: 4.7 mmol/L(n = 54)	1. There was no association between HPDS and serum potassium levels. 2. Higher HPD adherence was not associated with the odds of hyperkalaemia [OR 1.00 (95% CI 0.94– 1.07)].

HD, Hemodialysis; HPDS, healthy plant-based diet score; DASH, the dietary approaches to stop hypertension.

Khedr et al. (36) conducted a study with 400 MHD patients to investigate the prevalence of hyperkalemia in hemodialysis patients in Egypt. The study found that the pre-dialysis serum potassium of patients in the potassium-rich diet group (5.691 ± 0.462 mEq/L) was higher than that in the average potassium diet group (4.941 ± 0.376 mEq/L) and the low-potassium diet group (4.309 ± 0.321 mEq/L) (p < 0.01), and the incidence of hyperkalemia in hemodialysis patients was higher in the potassium-rich diet group than in the average or low-potassium diet group (p < 0.01) (36). Similarly, in exploring the association between dietary potassium intake and mortality in hemodialysis patients, Noori et al. (37) observed a positive correlation between dietary potassium and pre-dialysis serum levels (r=0.14, p<0.05), and the higher the potassium intake, the higher the risk of death.

However, in a study of dietary intake in 8043 adults with end-stage renal disease undergoing maintenance hemodialysis (DIET-HD) (38), dietary potassium intake was not associated with baseline serum potassium levels, dietary potassium was not associated with all-cause, cardiovascular, or noncardiovascular mortality, and consumption of fruits and vegetables was associated with a lower risk of all-cause death.

Likewise, Ramos et al. (39) investigated whether dietary potassium or specific food groups were linked with serum potassium in the presence of other risk factors, they discovered that potassium intake was directly related to fiber intake, as well as the intake of fruits, vegetables, and dairy products in the dialysis population’s diet, with no association founded between dietary potassium and protein intake or beans, meat, and eggs. Furthermore, this study also revealed that dietary potassium did not correlate with serum potassium in either HD or nondialysis CKD patients (r=-0.06, P=0.46; r=0.01, P=0.98) (39).

Additionally, Garagarza et al. (40) conducted a multi-center, cross-sectional study to investigate the association between the dietary approaches to stop hypertension (DASH) dietary pattern, which is high in potassium-rich foods, and serum potassium in hemodialysis patients, including 582 patients from 37 dialysis centers. No statistically significant association was found between serum potassium and dietary potassium intake in this study (r=0.080; p=0.060), which was consistent with the conclusion of Ramos et al. (39). This study showed no association between serum potassium and dietary potassium intake in the low potassium intake(≤3000 mg/d) and high potassium intake(>3000 mg/d) groups, and no apparent differences in serum potassium value between the two groups (40). According to the study, serum potassium levels were favorably connected with milk, eggs, beef, pork, chicken liver, fatty fish, squid, octopus, banana, canned fruit, wine, and coffee (P<0.05, respectively) in terms of food types. Additionally, boiled potatoes, cow and pork meat, white cabbage, apples and pears, cherries, yogurt, oranges, beans, peaches, tomatoes, and milk had larger positive correlations (r≥0.300) with dietary potassium consumption (P<0.001, respectively) (40).

In the Malnutrition, Diet, and Racial Disparities in Chronic Kidney Disease (MADRAD) study (415 participants), a higher risk of death was shown to be connected with reduced dietary potassium consumption, which also suggested that severe dietary potassium restriction may be detrimental for hemodialysis patients (41). Furthermore, González-Ortiz et al. (42) also emphasized that in a healthy plant-based diet (HPD), a higher healthy plant-based diet score (HPDS) was not connected with serum potassium levels or hyperkalemia (potassium >5.5 mEq/L) in hemodialysis patients and a higher HPDS was associated with a lower malnutrition inflammation score (MIS), indicating improved nutritional status. They concluded that in their observational study challenged the routinely advice to avoid fruits and vegetables in dialysis patients and emphasize the significance of conducting interventional studies that investigate the potential benefits and harms of liberalizing the diet of dialysis patients in term of the consumption of plant foods (42).

## Discussion

The above several studies have no consistent conclusions on the correlation between dietary potassium intake and serum potassium. The reason for the absence of a consistent positive correlation between serum potassium and dietary potassium could be related to the type of potassium-containing food and its content in other nutrients (40). In the DIET-HD trial, Bernier-Jean et al. (38) found that higher potassium intake from only whole plant sources was linked with a lower mortality risk, and that this association vanished after controlling for dietary groups, such as consumption of fruits and vegetables. Noori et al. (37) observed a positive correlation between dietary potassium and pre-dialysis serum levels in hemodialysis patients, in this study, dietary potassium was mainly derived from beef, chicken, Mexican food, burgers, beans, fresh fruits, fruit juices, fried potatoes, cheeseburgers and canned fruit, whereas in the DIET-HD study, the majority of potassium sources were vegetables, fresh fruit, red meat, potatoes, milk, and bread. Garagarza et al. (40) also found no association in their study of dietary patterns of high-potassium foods (DASH) and serum potassium in hemodialysis patients, considering that although there is no positive correlation between serum potassium and dietary potassium, this does not imply that high-potassium foods do not cause hyperkalemia in patients, due to differences in potassium bioavailability, distinct sources of dietary potassium (animal, plant, and potassium-based food additives) could contribute to elevations in different ways. In this study, the DASH dietary pattern emphasized consumption of plant-based foods with low potassium bioavailability (43, 44), with the exception of bananas, canned fruit, wine, and coffee, foods positively associated with serum potassium levels are primarily animal sources (milk, beef, pork and chicken liver, fatty fish, squid, and octopus).

Besides, an article on plant-based diets by Carrero et al. published in Nature Reviews Nephrology stated that for patients with chronic kidney disease(CKD) (17), current evidence shows that encouraging the adoption of plant-based diets has few hazards and has potential benefits for the primary prevention of CKD, and delaying progression in patients with CKD G3-5. This article recommended that limiting plant-based foods as a strategy for preventing hyperkalemia or malnutrition be done on an individual basis to avoid depriving CKD patients of the possibly positive effects of a plant-based diet (17). According to a European survey, only 4% of 8,078 hemodialysis patients took in four or more servings of fruits and vegetables per day, which is the recommended quantity for the general population (45). Besides, increasing fruit and vegetable consumption is associated with a lower risk of all-cause mortality, and there is an inverse correlation between consumption and non-cardiovascular mortality, which could be related to a lower risk of cancer death in hemodialysis patients (45). In a study of 81,013 hemodialysis patients investigating the association between pre-dialysis serum potassium levels and all-cause and cardiovascular mortality, potassium concentrations between 4.6-5.3 mEq/L were related to the lowest all-cause mortality (9). After correcting for confounding variables related to comorbidities and nutritional status, potassium levels ≥5.6 mEq/L were most strongly associated with higher mortality, although potassium levels <4.0 mEq/L were associated with increased mortality when malnutrition owing to inadequate dietary intake was taken into account (9).

Other than that, nutritional interactions must be taken into account when controlling dietary potassium intake and serum potassium levels. Plant-based foods with high potassium content include melons, citrus juice, and potatoes, it should be noted, however, that some food sources with high potassium content also contain high carbohydrates, which could stimulate insulin release and thus reduce the increase in plasma potassium concentration (6). Contrarily, animal products have higher potassium content but lower carbohydrate content, which may lead to higher plasma potassium levels after intake, therefore, not all potassium-rich foods are likely to result in similar increases in serum potassium concentrations (6). Garagarza et al. (40) noted that carbohydrate-rich and potassium-rich foods may have less effect on serum potassium than low-carbohydrate and high-potassium foods, because there are other nutrients in food, such as fiber, affect potassium distribution and excretion, and increased carbohydrate intake contributes to high fiber intake, which in turn causes potassium excretion (46). Besides, when increased potassium intake sufficiently increases plasma potassium concentrations, aldosterone also enhances potassium excretion in the distal colon (47), and adaptation of this function may be very crucial, especially when renal function is impaired (48). Hayes et al. (49) demonstrated that fecal potassium excretion was three times higher in hemodialysis compared with normal controls and could even reach 80% of dietary potassium in some cases, in addition to fecal potassium content being proportional to dietary potassium intake consumption and fecal weight. Since the digestive system is another route for excreting potassium (48, 50), it’s crucial to consume enough fiber in the diet. For example, the DASH diet includes plenty of plant foods, which are high in fiber and can increase fecal excretion of potassium through stimulating intestinal peristalsis (46, 51, 52). On the other hand, considering the relatively high incidence of constipation in HD patients (53%) (53), infrequent bowel movements, rather than dietary potassium load, may be a major driver of hyperkalemia in HD patients (46). In a review of plant-based low-protein diets in the conservative management of patients with CKD, it was shown that meat consumption increases the production of nitride-containing end products, exacerbates uremia, and may lead to constipation due to inadequate fiber intake, thereby increasing the risk of hyperkalemia (54). In contrast, a plant-based diet may lower gut-derived uremic toxins by increasing intake of fiber and regulating the intestinal microbiota (55). In one study, increasing dietary fiber intake for 6 weeks in hemodialysis patients could reduce free plasma indoxyl sulfate (a uremic toxin produced by the breakdown of aromatic amino acids by intestinal microbiota) levels by 29% (56). On the other hand, a plant-based diet of wholesome fruits and vegetables reduces the likelihood of potassium-containing additives commonly found in meat products (57, 58). This dietary pattern also includes additional factors that may assist prevent serum potassium increases, such as a high consumption of alkaline foods (fruits and vegetables), which may enhance intracellular potassium transport, particularly in the context of metabolic acidosis (40).

Therefore, when evaluating the effect of diet on serum potassium, it is important to consider not only the potassium content of foods, but also the type of food and other nutrient content. Furthermore, excessive dietary potassium restriction may not be beneficial for survival in hemodialysis patients, and further research is required to explore the ideal dietary potassium intake for this population. Some studies have advocated for moderate potassium restriction in hemodialysis patients, or relaxation of potassium intake with the prescription of potassium binders (59, 60).

## Analysis of other common influencing factors of predialysis serum potassium

Angiotensin converting enzyme inhibitor/Angiotensin receptor blocker (ACEI/ARB), Spironolactone, β-Receptor Blockers are common drugs that affect serum potassium fluctuations. Taking ACEI/ARB in patients with end-stage renal disease can not only cooperate with renin-angiotensin-aldosterone system (RAAS) related blood pressure increase, but also improve ventricular remodeling, which can effectively reduce the mortality of MHD patients by reducing blood pressure and reversing left ventricular hypertrophy (LVH) (61). ACEI/ARB drugs affect serum potassium levels by inhibiting aldosterone secretion, increasing prostaglandin or bradykinin synthesis, and decreasing potassium ion excretion. Knoll et al. (62) studied the risk of hyperkalemia in patients with renin-angiotensin system blockade and chronic hemodialysis, they found that the use of ACEIs/ARBs was significantly associated with an increased risk for hyperkalemia (p <0.05). However, other clinical trials and meta-analysis evaluating RAAS blockers in hemodialysis populations have shown no difference in serum potassium or frequency of hyperkalemia between intervention and control groups (63–67). It has also been reported that the use of ACEIs/ARBs had no effect on hyperkalemia in MHD patients, where neither monotherapy (ACEIs or ARBs) nor combination therapy (ACEIs plus ARBs) was associated with an excess risk of hyperkalemia in MHD patients (68). Similarly, clinicians tend to worry that spironolactone would raise the risk of hyperkalemia in hemodialysis patients and limit its usage due to its “sodium excretion and potassium retention” (69, 70). The addition of low-dose of spironolactone or combined with conventional treatment is safe and will not significantly increase serum potassium levels, but more importantly, can improve LVH by lowering left ventricular mass index (LVMI) and raising left ventricular ejection fraction (LVEF), according to systematic reviews and meta-analyses of several studies analyzing the effects and safety of spironolactone on the cardiovascular system in the routine treatment of hemodialysis patients (71–76). In contrast to controls, spironolactone increased the frequency of moderate hyperkalemia (6.0-6.5 mmol/L), but not severe hyperkalemia (≥6.5 mmol/L), according to a study on the safety and efficacy of the drug in hemodialysis patients (77). With regards to β-receptor blockers, because nonselective β-receptor blockers (such as propranolol) can interfere with Na+-K+-ATPase on the cell membrane and prevent potassium ions from entering the cell, serum potassium levels rise (78). Some studies have demonstrated that β-blocker therapy could be used safely in hemodialysis patients, with severe hyperkalemia occurring in only a minority of patients (36, 79). In contrast, serum potassium levels were observed to correlate with spironolactone, ACEIs, and β-blocker intake in a study by Muschart X et al. (80). Furthermore, insulin resistance, race, and gender variances can all have an impact on serum potassium levels (81–83). Among the studies included in this paper, Khedr et al. (36) found no significant association between serum potassium and ACEls, β-blockers, or diabetes, and Ramos et al. (39) observed no notable distinction in serum potassium between the normokalemic and hyperkalemic groups in dialysis patients treated with β-blockers (P=0.79).

At present, there are still differing conclusions regarding whether aforementioned medications can affect the serum potassium level of hemodialysis patients. Since the renal potassium excretion function is nearly lost in MHD patients, dialysis is the primary means of controlling serum potassium excretion. Some researchers believe that hyperkalemia may not be a major concern in hemodialysis patients since dialysis, rather than renal tubular function, controls the factors affecting serum potassium in dialysis patients (84, 85). Thus, with the adequacy of dialysis, close monitoring of serum potassium, and timely adjustment of dialysate composition, whether taking ACEIs/ARBs, β-blockers, mineralocorticoid antagonists, or different residual renal function, may not have a significant effect on serum potassium levels in hemodialysis patients.

## Conclusion

Based on the current small observational studies, we discovered that in cases where dialysis adequacy was ensured and specific dietary patterns (such as the above-mentioned plant-based diet) were followed, there was less or even no correlation between dietary potassium intake and serum potassium in hemodialysis patients; excessive dietary potassium restriction may not benefit the survival of hemodialysis patients. Additionally, when assessing the effect of diet on serum potassium, researchers should not only focus on the potassium content of foods, but also consider the type of food and the content of other nutrients. Meanwhile, we admit that publication bias may be present in this result attributed to the several positive small center series. Therefore, more large-scale, multi-center clinical trials are needed to provide high-quality evidence support, and we also need to further explore the dietary patterns and optimal daily dietary potassium intake that are beneficial to hemodialysis patients.

## Author contributions

ZS: Methodology, Writing – original draft. JJ: Writing – original draft. GL: Methodology, Writing – review & editing. RL: Methodology, Writing – review & editing. ZL: Supervision, Writing – review & editing. YS: Supervision, Writing – review & editing. ZC: Supervision, Writing – review & editing.
